# Lessons Learned in the Management of Eclampsia: A Retrospective Observational Study in Pregnant Women

**DOI:** 10.7759/cureus.83015

**Published:** 2025-04-25

**Authors:** Rajasri Yaliwal, Shreedevi Kori, Preeti S Malapure, Kota Sai Meghana, Sushmitha Reddy

**Affiliations:** 1 Obstetrics and Gynaecology, Shri B M Patil Medical College Hospital and Research Centre, Vijayapura, IND

**Keywords:** convulsion, eclampsia, magnesium sulfate, maternal outcomes, perinatal outcome

## Abstract

Background

Eclampsia is a critical obstetric emergency associated with significant maternal and fetal mortality and morbidity. This retrospective observational study assesses the clinical characteristics, management strategies, and findings in eclamptic patients, emphasizing lessons learnt from treatment delays and therapeutic interventions.

Aims

The study aimed to determine perinatal outcomes in eclamptic women and to evaluate perinatal outcomes based on the interval between the initial convulsion and delivery, as well as the duration of treatment before delivery and delivery methods.

Study setting and design

Eclamptic women who met the inclusion criteria and were admitted from January 1, 2012, to December 31, 2024, to the labor ward at BLDE (DU) Shri B M Patil Medical College Hospital and Research Centre in Vijayapura, Karnataka, India, were included in this study. Investigation findings and medical data were gathered and assessed.

Results

The study included 192 pregnant women having eclampsia, all beyond 28 weeks of gestation, meeting specified inclusion and exclusion criteria. A total of 192 babies were delivered, with 58 perinatal deaths (30.2%) recorded. Obstetric analysis revealed that primigravida patients constituted the majority, reinforcing their higher risk profile. Perinatal mortality was raised in individuals with systolic blood pressure (BP) of ≥160mm Hg, diastolic of ≥110mm Hg, newborns of birth weight less than 2 kg, and urine albumin levels exceeding 2+. Perinatal mortality was comparatively low when provided within six hours of convulsion and starting medical care. The cesarean section rate was high, reflecting the need for rapid stabilization.

Conclusion

This study highlights that early and appropriate use of medical management coupled with decisive delivery planning results in high fetal viability and acceptable maternal outcomes. The predominance of primigravida and the high cesarean rates suggest that eclampsia management protocols require continuous refinement to improve response times and further enhance fetomaternal safety. Emphasis on early recognition and rapid intervention remains essential in reducing morbidity and mortality associated with this obstetric emergency.

## Introduction

In India, eclampsia affects approximately one in 500 to one in 2,000 pregnancies, with varying prevalence across different regions and healthcare settings. Approximately 1.4% of deliveries are affected, and maternal death rates range from 2% to 30%. In developing nations, rates of perinatal death due to eclampsia vary from 30% to 50%, reflecting disparities in healthcare access [1]. Seizures are typically generalized tonic-clonic (GTCS), lasting approximately one minute, followed by confusion or coma. Seizures occur postnatal in 44% of cases, antepartum in 38%, and intrapartum in 18%. Before convulsions, women commonly experience symptoms such as headaches (the most prevalent symptom), nausea, blurred vision, vomiting, confusion, and photophobia, though some may present without any preceding symptoms. Despite recent advancements in technology, radiology, and surgical and medical domains, utilization of antihypertensive and prophylactic medications, along with vigilant monitoring, eclampsia remains a significant healthcare issue in pregnant women, adversely impacting both maternal and fetal outcomes [1-3].

Perinatal morbidities in eclampsia vary from 5% to 11.8% in developed nations and reach 40% in developing nations. In premature delivery, intrauterine fetal growth limitations and birth asphyxia are often linked to the condition and increased perinatal mortality [4]. In approximately 16% of eclampsia patients, hypertension may be unreported. Proteinuria may be absent in approximately 14% of eclampsia patients, whereas significant proteinuria may be present in approximately 50% of cases [5,6]. The ubiquitous accessibility and easy availability of healthcare facilities for nearly all pregnant women can facilitate the prompt detection and prevention of eclampsia, thereby reducing perinatal and maternal morbidity and mortality, which has been linked to a decline in eclampsia incidence in developing countries [7,8].

This study aimed to explore perinatal outcomes in women with eclampsia and identify factors influencing these outcomes to implement preventive strategies. Furthermore, the study’s retrospective design allowed for an in-depth analysis of time intervals related to the management of eclampsia, highlighting systemic delays that can have profound implications for both maternal and neonatal outcomes.

## Materials and methods

This study employed a retrospective observational design and was conducted at a tertiary care hospital that functions as a referral center for high-risk obstetric cases. The hospital is equipped with specialized maternal and neonatal intensive care units, allowing for the comprehensive management of complex obstetric emergencies. Data for this study were extracted from the medical records of patients who met the inclusion criteria. These records were reviewed to obtain relevant clinical and demographic information. Ethical clearance for the study was granted by the Institutional Ethics Committee, ensuring compliance with the Declaration of Helsinki and the national ethical guidelines (Reference no. BLDE[DU]/IEC/559/2021-22).

**Figure 1:**
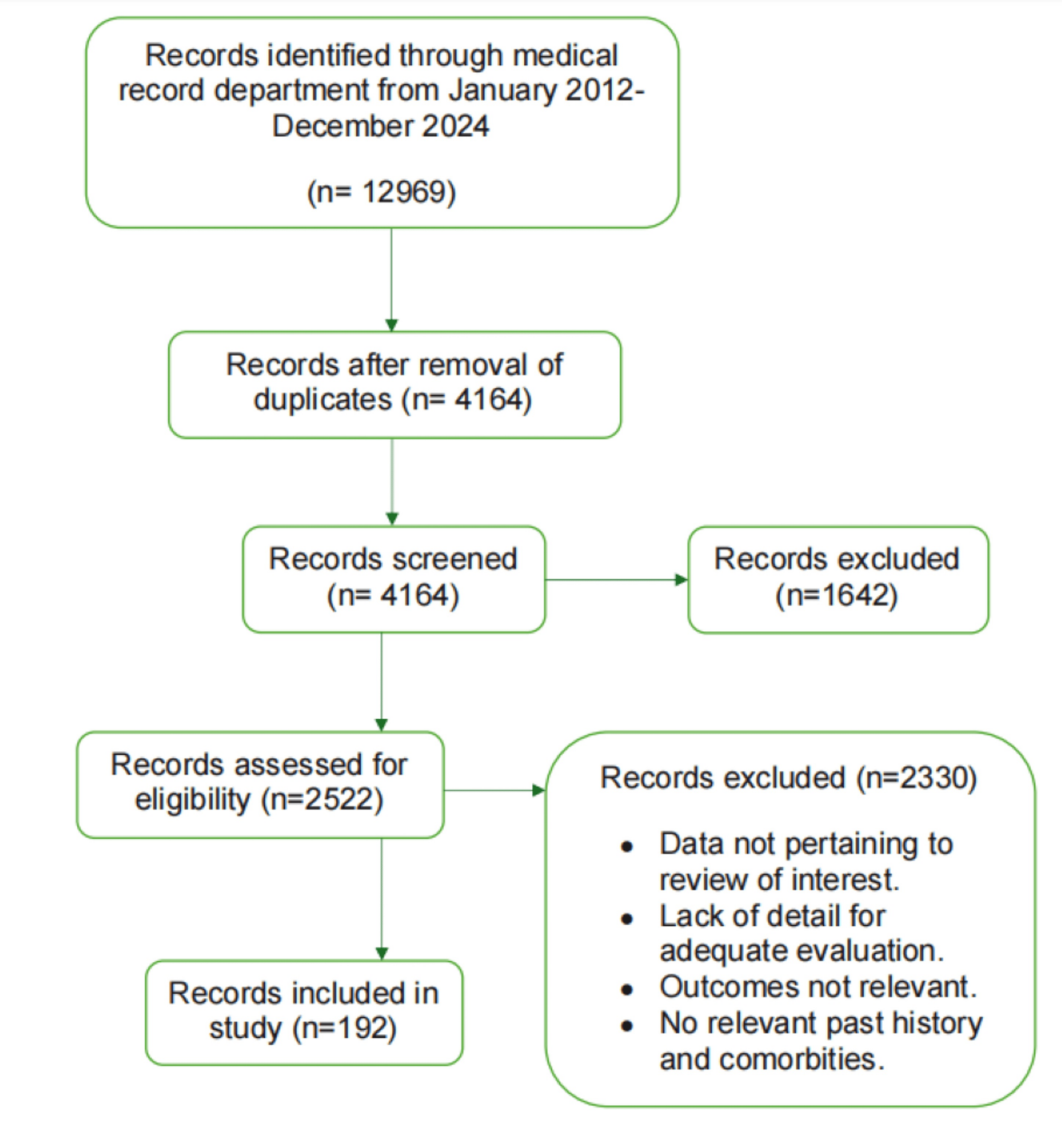
Selection protocol of study participants

The study included all pregnant women aged 18 years and above who were admitted with intrapartum or antepartum eclampsia to the labor ward of BLDE (Deemed to be University) Shri B M Patil Medical College Hospital and Research Center, located in Bijapur, Karnataka, India. The study period extended from January 1, 2012, to December 31, 2024, encompassing all relevant retrospective cases within this timeframe (Figure 1).

Participants were included in the study if they were 18 years or older, had a confirmed diagnosis of intrapartum or antepartum eclampsia, and were admitted to the labor ward during the study period. Patients were excluded if they had pre-existing chronic medical conditions such as chronic hypertension or renal disease, were known cases of diabetes mellitus, or were referred from other healthcare facilities where details regarding primary management and clinical information were insufficient or unavailable.

Each file was carefully examined to extract relevant clinical and demographic information, including age, parity, gestational age at presentation, blood pressure (BP) readings, presence of proteinuria, number of convulsions, timing of delivery in relation to seizures, mode of delivery, maternal complications, and neonatal outcomes. Data collection was carried out using a structured proforma to ensure consistency and minimize bias. Only cases with complete documentation were included, and all extracted data were anonymized before analysis.

Statistical analysis was conducted using the IBM SPSS Statistics for Windows, Version 26 (Released 2019; IBM Corp., Armonk, New York, United States). Data were initially entered and organized in Microsoft Excel before being imported into SPSS for further analysis. Descriptive statistics were used to summarize demographic and clinical characteristics. Categorical variables were expressed as frequencies and percentages. Comparative analyses were performed to evaluate differences between groups using the Chi-square test for categorical variables. All statistical tests were two-tailed, and a p-value of <0.05 was considered statistically significant.

## Results

Out of 12,969 deliveries that occurred at the hospital during the study period, our study included 192 patients who met the inclusion criteria. All 192 patients presented with either intrapartum or antepartum eclampsia. Management of all cases involved the administration of magnesium sulfate using Pritchard’s regimen. Among these, 48 (25.0%) were booked cases, while 144 (75.0%) were unbooked. A total of 98 (51.0%) patients were referred from other healthcare facilities. Additionally, 101 (52.6%) patients lacked regular antenatal check-ups. Gravida distribution revealed that 142 (73.95%) were primigravida, 30 (15.6%) were second gravida, and 20 (10.4%) were third gravida or more. Age-wise, the most affected group was 18-20 years with 79 (41.1%) patients, followed by 76 (39.6%) aged 21-25 years, 21 (10.9%) aged 26-30 years, and 16 (8.3%) above 30 years.

Additional baseline characteristics showed a mean maternal age of 22.7 ± 3.9 years and a mean gestational age of 35.8 ± 3.3 weeks. Comorbidities were limited; thyroid disorders were observed in 31 (16.1%) patients, pallor in 46 (24.0%), and edema in 141 (73.4%). The mean systolic BP was 156.8 ± 22.4 mmHg, the diastolic BP was 102.0 ± 14.7 mmHg, and the average hemoglobin was 11.7 ± 1.9 g/dL. Mean uric acid was 7.0 ± 1.8 mg/dL. Regarding the type of eclampsia, 147 (76.56%) had antepartum and 45 (23.44%) had intrapartum eclampsia (Table 1).

**Table 1:** Type of eclampsia

Type of eclampsia	Frequency	Percentage
Antepartum	147	76.56
Intrapartum	45	23.44
Total	192	100

Gestational age assessment showed that 102 (53.12%) patients delivered preterm (<37 weeks), while 90 (46.87%) had full-term pregnancies. This finding underscores the risk of preterm delivery in eclampsia and its implications for neonatal outcomes.

Among the 192 deliveries, 87 (45.31%) were conducted via normal vaginal delivery, and 105 (54.69%) underwent lower segment cesarean section (LSCS). The most common indication for LSCS was fetal distress, followed by cephalopelvic disproportion, previous cesarean, nonprogression of labor, failed induction, and, least common, malpresentation (Table 2).

**Table 2:** Indications for LSCS LSCS: lower segment cesarean section; TOLAC: trial of labor after cesarean

LSCS indication	Frequency	%
Fetal distress	26	24.77
Cephalopelvic disproportion	22	20.95
Previous cesarean not willing for TOLAC	19	18.10
Nonprogression of labor	19	18.10
failure of induction	16	15.23
Malpresentation	3	2.85
Total	105	100

A total of 192 babies were delivered. Perinatal outcomes included 15 intrauterine deaths, 25 fresh stillbirths, and 18 early neonatal deaths. Only 134 (69.79%) neonates were discharged healthy. Perinatal mortality was not significantly associated with the number of convulsions, highest among those with 6-10 episodes, and none reported in patients with >10 convulsions (Table 3).

**Table 3:** Association between the number of convulsions and perinatal death Frequency and percentages were calculated. A Chi-square test was applied, and a p-value less than 0.05 was considered significant

No. of convulsions	No of patients	Perinatal death	%
≤5	92	26	28.26
6 to 10	97	32	32.99
>10	3	0	0
Chi-sqaure value	1.817		
p-value	0.403		

Of the 75 neonates with birth weight ≥2.5 kg, only five (6.66%) perinatal deaths occurred. Conversely, perinatal mortality was highest among neonates weighing 1-1.5 kg, where 17 out of 23 (73.91%) died (Table 4).

**Table 4:** Association between birth weight and perinatal mortality Frequency and percentages were calculated. A Chi-square test was applied, and a p-value less than 0.05 was considered significant

Birth weight	No. of patients	Perinatal death	%
≥2.5 kg	75	5	6.66
2-2.5 kg	44	28	63.63
1.5-2 kg	40	8	20
1-1.5 kg	23	17	73.91
Chi-square value	63.71		
p-value	<0.0001		

Time elapsed between the initial convulsion and delivery was another crucial factor. Perinatal mortality increased with delay in delivery after the first convulsion, rising from 16 (20.5%) within six hours to 20 (54.05%) beyond 18 hours (Table 5).

**Table 5:** Association between convulsion-to-delivery interval and perinatal death Frequency and percentages were calculated. A Chi-square test was applied, and a p-value less than 0.05 was considered significant

Convulsion-to-delivery interval	No. of patients	Perinatal death	%
<6 hours	78	16	20.51
6 to 11 hours	50	12	24
12-17 hours	27	10	37.03
≥18 hours	37	20	54.05
Chi-square value	7.35		
p-value	0.061		

A similar trend was noted regarding the interval between treatment initiation and delivery. Among 88 patients who delivered within six hours of starting treatment, 14 (15.9%) perinatal deaths occurred. Delays of 6-11 hours and ≥12 hours led to perinatal mortality of 27 (47.36%) and 17 (36.17%), respectively (Table 6).

**Table 6:** Association between treatment-to-delivery interval and perinatal death Frequency and percentages were calculated. A Chi-square test was applied, and a p-value less than 0.05 was considered significant

Treatment-to-delivery interval	No. of patients	Perinatal death	%
<6 hours	88	14	15.9
6 to 11 hours	57	27	47.36
≥12 hours	47	17	36.17
Chi-square value	17.288		
p-value	0.0001		

Dipstick urine analysis showed that patients with ≥3+ albuminuria had higher perinatal mortality (10/22, 45.45%) compared to those with trace or +1 levels (Table 7).

**Table 7:** Association between urine albumin and perinatal death Frequency and percentages were calculated. A Chi-square test was applied, and a p-value less than 0.05 was considered significant

Albumin	No. of patients	Perinatal death	%
0	26	8	30.76
+1	94	18	19.15
+2	43	19	44.18
+3	22	10	45.45
+4	7	3	42.85
Chi-square value	12.3988		
p-value	0.0146		

BP analysis showed elevated perinatal mortality in patients with a systolic BP of ≥160 mmHg (33/71, 46.47%) and diastolic BP of ≥110 mmHg (16/65, 24.61%) (Table 8).

**Table 8:** Association between blood pressure and perinatal deaths Frequency and percentages were calculated. A Chi-square test was applied, and a p-value less than 0.05 was considered significant

BP	No. of patients	Perinatal death	%
Systolic BP ≥ 160 mm of Hg	71	33	46.47
Systolic BP < 160 mm of Hg	30	5	16.66
Diastolic BP ≥ 110 mm of Hg	65	16	24.61
Diastolic BP < 110 mm of Hg	26	4	15.38
Ci-square value	15.1989		
p-value	0.0016		

The mode of delivery influenced perinatal mortality. Among 87 vaginal deliveries, 34 (39.0%) resulted in perinatal deaths. In contrast, 24 (22.85%) deaths occurred among the 105 cesarean deliveries (Table 9).

**Table 9:** Association between mode of delivery and perinatal deaths Frequency and percentages were calculated. A Chi-square test was applied, and a p-value less than 0.05 was considered significant

Type of delivery	No. of patients	Perinatal death	%
Vaginal delivery	87	34	39
Cesarean delivery	105	24	22.85
Chi-square value	5.9395		
p-value	0.0148		

Uric acid levels were also associated with outcomes: Patients with uric acid ≥7.1 mEq/dL had the highest perinatal mortality (12/27, 44.44%) (Table 10).

**Table 10:** Association between uric acid levels and perinatal deaths Frequency and percentages were calculated. A Chi-square test was applied, and a p-value less than 0.05 was considered significant

Uric acid level (mEq/dl)	No. of patients	Perinatal death	%
<5.5	55	10	18.18
5-6.7	110	36	32.72
≥7.1	27	12	44.44
Chi-square value	6.6997		
p-value	0.0351		

At five minutes post-birth, Apgar scores were normal in 52 (38.8%) neonates, intermediate in 57 (43.8%), and low in 25 (18.65%) newborns. Among neonatal complications, birth asphyxia was most common, affecting 34 (17.7%) neonates, followed by prematurity in 30 (15.6%) and respiratory distress syndrome (RDS) in 24 (12.5%) (Table 11).

**Table 11:** Neonatal complications IUGR: intrauterine growth restriction Frequency and percentages were calculated

Neonatal complications	No. of cases	%
Hyperbilirubinemia	22	11.4%
Respiratory distress syndrome	24	12.5%
Prematurity	30	15.6%
Meconium aspiration	18	9.4%
IUGR	6	3.1%
Birth asphyxia	34	17.7%

These findings comprehensively highlight the maternal and neonatal risks associated with eclampsia and form the basis for outcome-based evaluation and targeted management strategies.

## Discussion

This retrospective observational study, conducted over a 12-year period at a tertiary care center, aimed to evaluate the clinical profile, management, and perinatal outcomes associated with eclampsia. With an incidence rate of 1.48%, our findings highlight that eclampsia continues to pose a significant obstetric emergency, particularly in low-resource settings. Despite advancements in prenatal care and hypertensive disorder management, the condition remains a major contributor to maternal and perinatal morbidity and mortality. By analyzing a cohort of 192 eclamptic women, we were able to assess critical risk factors, treatment efficacy-particularly the use of magnesium sulfate-and outcome determinants such as timing of delivery, antenatal care status, and associated complications like proteinuria and severe hypertension. These insights are essential for improving early detection, risk stratification, and timely intervention strategies in similar clinical environments.

The incidence of eclampsia in our study was 1.48%, which aligns closely with findings from other Indian studies. Kaur et al. reported an incidence of 1.6% [9], Dhanapal et al. reported 1.35% [10], Swain et al. observed 1.39% [11], while Das et al. reported a higher incidence of 2.67% [12]. In contrast, a significantly lower incidence of 0.17% was reported by Doley et al. [13] 

In developed countries, the incidence of eclampsia ranges from 1.6 to 10 per 10,000 pregnancies, whereas in developing nations, it ranges from 50 to 151 per 10,000 deliveries [14]. This disparity reflects differing standards in prenatal care, healthcare infrastructure, and accessibility. In low-resource settings, maternal and perinatal morbidity and mortality associated with eclampsia remain significantly higher. Magnesium sulfate has proven highly effective in reducing seizure recurrence; with its administration, eclamptic seizures occur in less than 0.6% of women, compared to 2% in those who do not receive it [14]. In our study, no cases of magnesium sulfate toxicity were observed, suggesting the effectiveness and safety of our therapeutic protocols, though caution is still necessary in high-risk populations.

Out of 192 eclampsia cases, 142 were primigravida, and 79 patients were under 20 years of age, primarily aged 18-20 years. Our results align with the established understanding that eclampsia is more prevalent among primigravida. This trend is supported by research from Sibai and Cunningham [15] and further validated by Conde-Agudelo’s study on Latin American populations and Saxena et al.'s findings in India, both of which identified nulliparity as an independent risk factor for severe pre-eclampsia and eclampsia [16,17].

A significant majority (75%) of the patients were unbooked, with 73.95% being primigravida. Our findings align with those of Katz et al., who reported a 70% primigravida rate among eclamptic patients [18]. These results highlight the consequences of poor antenatal awareness and healthcare access. Furthermore, 51% of our patients were referred from peripheral hospitals, often in critical condition.

Unbooked status correlates with increased incidence of hypertensive complications, undetected anemia, and poor perinatal outcomes. Contributing factors include illiteracy, sociocultural barriers, financial constraints, limited transportation, and inadequate telecommunication infrastructure [5]. Gandhi et al. reported that 72.6% of eclampsia and pre-eclampsia cases in rural Gujarat occurred in unbooked women, reinforcing our findings [19].

Preterm deliveries were observed in 53.12% of cases, reflecting the known association between eclampsia and prematurity. Tuffnell et al. reported similar findings (65.3%) [20]. Major contributors to perinatal morbidity and mortality included birth asphyxia, intrauterine growth restriction (IUGR), RDS, prematurity, and inadequate prenatal care.

Our study found that patients experiencing more than five convulsions exhibited significantly higher perinatal mortality, underscoring the critical need for timely intervention. This finding is consistent with Dhananjay et al., who also reported poor perinatal outcomes in patients with frequent convulsions [21]. Early delivery, especially within six hours of convulsions, was associated with significantly better perinatal outcomes. Previous research supports this observation, emphasizing the critical importance of timely delivery to reduce perinatal mortality [22].

Proteinuria levels of ≥2+ and elevated blood pressure (systolic ≥160 mmHg and diastolic ≥110 mmHg) were associated with higher perinatal mortality, which aligns with findings from other studies [23]. Mustaphi et al. also found increased perinatal mortality with maternal serum uric acid levels above 6 mg/dL, corroborating our study’s observations [24].

Although Apgar scores are widely used, the American Academy of Pediatrics and the American College of Obstetrics and Gynecology caution that one- and five-minute Apgar scores alone are insufficient to determine the cause or prognosis of asphyxia [25].

Cesarean section has revolutionized obstetrics, contributing to reduced neonatal morbidity and mortality. In our study, C-section outcomes were comparable to vaginal deliveries. Similar results were documented in studies highlighting cesarean delivery in optimizing outcomes in high-risk pregnancies [26,27].

One of the major strengths of this study is the inclusion of 192 eclampsia cases, which enhances the statistical power and reliability of the findings. The data were collected over 12 years, from 2012 to 2024, reducing the influence of short-term variations and providing a more stable and comprehensive understanding of eclampsia trends. Conducting the study in a tertiary referral center added further depth, as the facility manages a wide range of high-risk and complex obstetric cases, allowing for the evaluation of diverse clinical presentations. Additionally, the study employed a multifactorial analysis of clinical parameters such as time from convulsion to delivery, blood pressure levels, proteinuria, and birth weight, thereby offering valuable insights into the determinants of perinatal outcomes in eclampsia.

Despite its strengths, the study has several limitations that must be acknowledged. Being a retrospective study, it relies on the accuracy and completeness of existing medical records, which may include inconsistencies or missing data, potentially affecting the comprehensiveness of our findings The single-center design may also limit the generalizability of the findings, particularly to rural or under-resourced healthcare settings where the level of care and availability of emergency interventions differ significantly. Moreover, the lack of a normotensive control group restricts comparative risk analysis, which could have further contextualized the outcomes observed. Variations in clinical practice, staff expertise, and medication availability over the 12 years may also have introduced confounding factors that could affect the consistency of the results.

## Conclusions

Pre-eclampsia and eclampsia are significant contributors to maternal and fetal morbidity and mortality. Although complete prevention may not be achievable, early recognition of warning signs is crucial to avoid potentially fatal consequences. Our study shows that fewer prenatal visits correlate with an increased risk of complications; therefore, improved awareness and access to care are needed to enhance antenatal services. Early recognition of eclampsia, prompt initiation of treatment with magnesium sulfate, and delivery within six hours of seizure onset are essential for improving perinatal outcomes. Strengthening antenatal care, particularly among primigravida and unbooked patients, is crucial to reducing preventable morbidity and mortality.
